# Supplementary material for: Neuronal toll-like receptor-4 regulation of matrix metalloproteinase-9 activity mediates dentate circuit dysfunction after traumatic brain injury
Source: J Neuroinflammation. 2026 Jun 3;23:271. doi: 10.1186/s12974-026-03890-4 (PMC13474604; doi:10.1186/s12974-026-03890-4)
Supplement: Supplementary file 1 [file 12974_2026_3890_MOESM1_ESM.pdf]

Neuronal Toll-like Receptor-4 regulation of Matrix Metalloproteinase-9 Activity Mediates Dentate Circuit Dysfunction after Traumatic Brain Injury

Deepak Subramanian[1#], Erick Contreras[1], Laura Dovek[2], Razieh Jaberi[1], Emmanuel Green[1], Ysabelle K Lao[1], Iryna M Ethell[2] and Vijayalakshmi Santhakumar[1#]

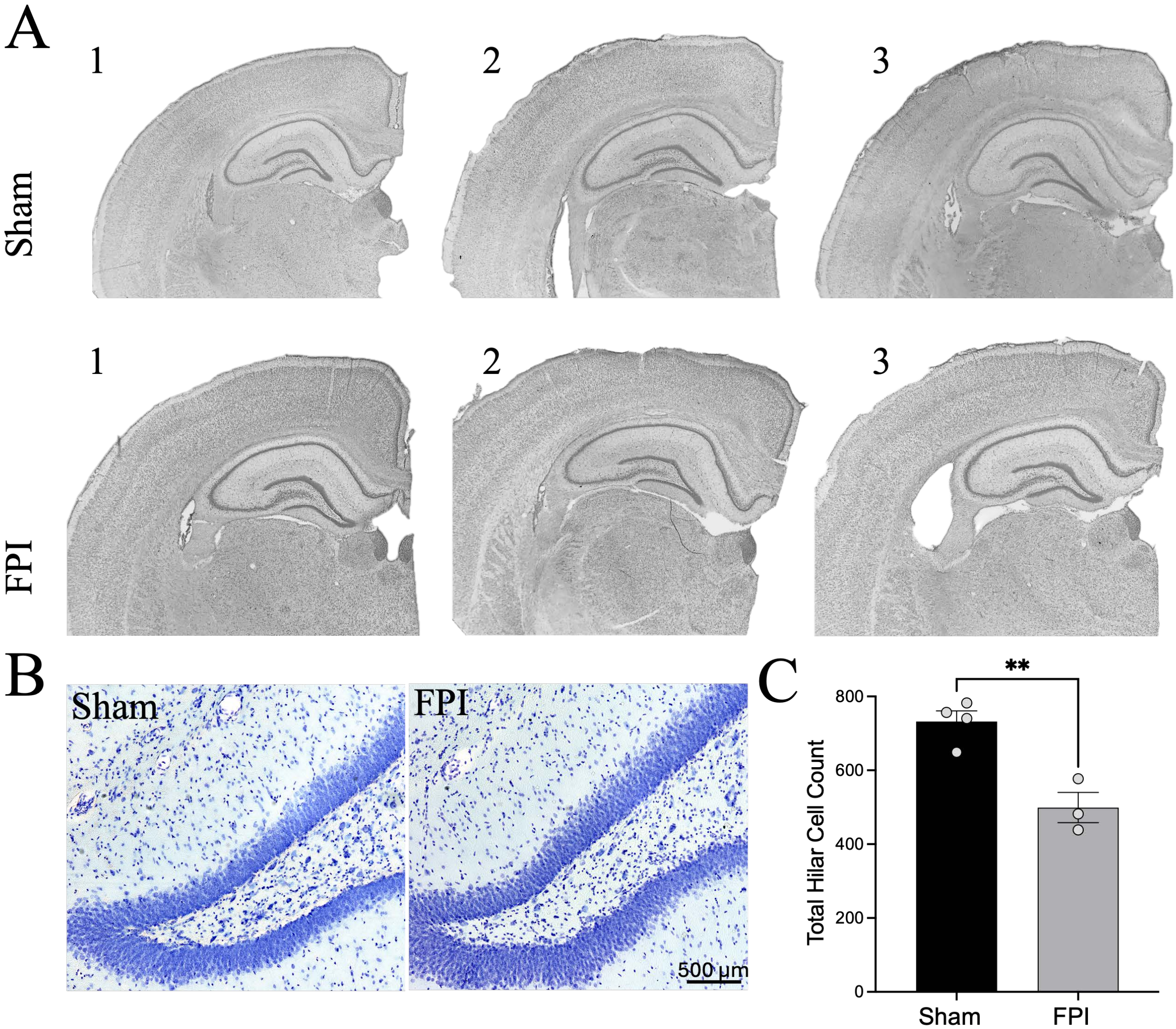

Supplementary Figure 1: Absence of overt histopathological changes and consistent dentate gyrus hilar cell loss after Lateral Fluid Percussion Injury. A) Representative images of Nissl-stained slices from 3 sham and 3 FPI rats 1-month post-injury showing no gross structural damage. B) Representative Nissl-stained images showing reduced hilar neurons in injured animals. C) Summary plot shows injury-induced decrease in hilar neuron count. n = 4 sham and 3 FPI rats. ** indicates P<0.01 by unpaired t-test.

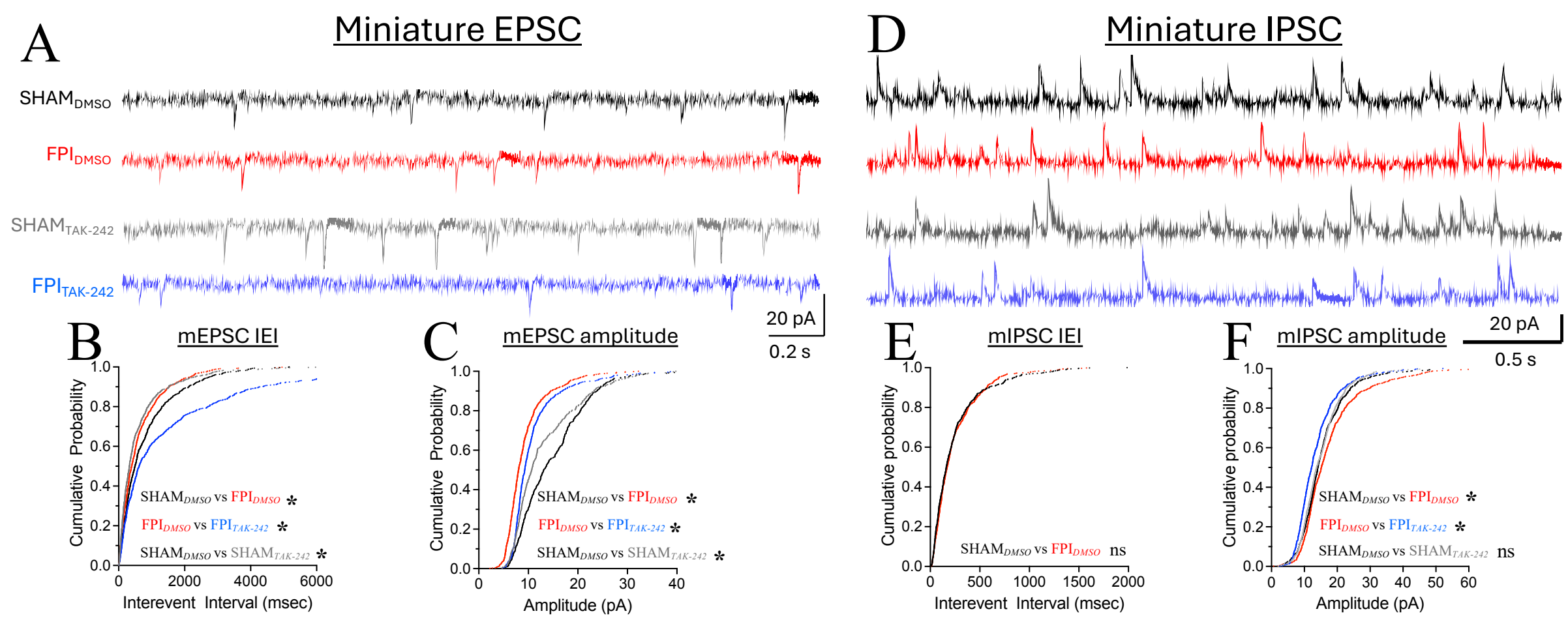

Supplementary Figure 2: ***TLR4 signaling drives changes in miniature excitatory and inhibitory inputs to DGCs after brain injury.*** A) Representative granule cell mEPSC traces from vehicle treated sham controls (black), vehicle treated FPI (red), TLR4 inhibited sham (grey) and TLR4 inhibited FPI (blue) rats. B-C) Cumulative probability plots of mEPSC interevent intervals (B) and amplitude (C) in experimental groups. D) Representative mIPSC traces from granule cells in vehicle treated sham controls (black), vehicle treated FPI (red), TLR4 inhibited sham (grey) and TLR4 inhibited FPI (blue) rats. E-F) Cumulative probability plots of mIPSC interevent intervals (E) and amplitude(F) in vehicle treated and TLR4 inhibited sham and injured rats. n = 7 cells from 3 rats/group *indicates P<0.05 by Holm-Bonferroni corrected K.S. test.

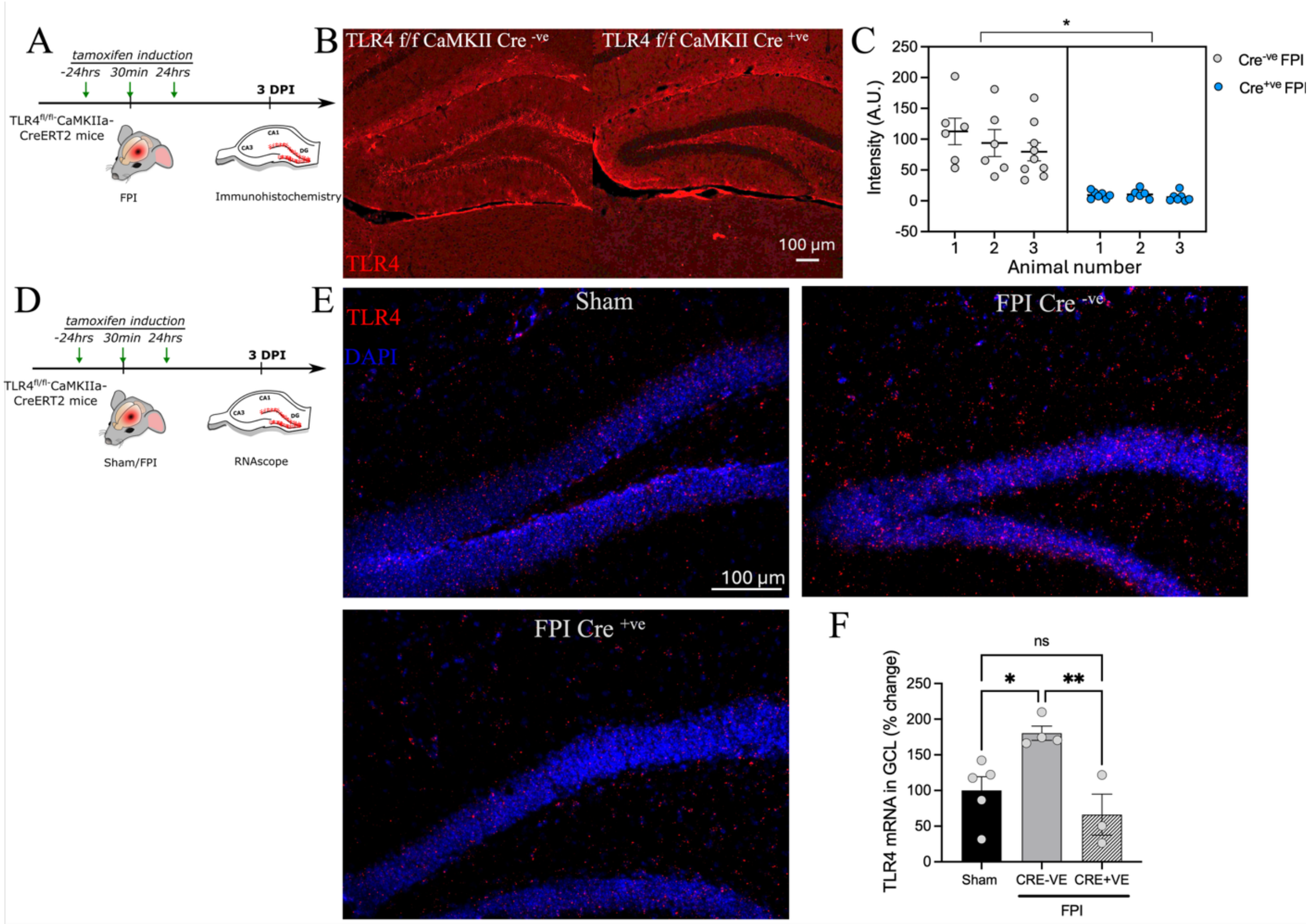

Supplementary Figure 3: *Validation of cell-type specific TLR4 deletion in DG glutamatergic neurons.* A) Experimental timeline for evaluating TLR4 expression after tamoxifen induction in $TLR4^{fl/fl}$: CaMKIIa-CreERT2 mice and littermate Cre^{-ve} mice. B) Representative images of TLR4 expression in the DG of tamoxifen treated to Cre^{-ve} and $TLR4^{fl/fl}$: CaMKIIa-CreERT2 mice 3 days after injury. C) Summary plot of TLR4 expression in experimental groups. * indicates $P<0.05$ by nested t-test based on n = 5-6 slices / mouse (indicated as animal number on x-axis) from 3 mice/group. D) Experimental timeline for evaluating TLR4 mRNA after tamoxifen induction in $TLR4^{fl/fl}$: CaMKIIa-CreERT2 mice and littermate Cre^{-ve} mice. E) Representative images of TLR4 mRNA expression in the DG of a wild-type sham mouse, tamoxifen treated to Cre^{-ve} and $TLR4^{fl/fl}$: CaMKIIa-CreERT2 mice 3 days after injury and a negative control. F) Summary plot of TLR4 mRNA

expression in the granule cell layer (GCL) in experimental groups. * indicates $P<0.05$ and ** indicates $P<0.01$ by OW-ANOVA, based on data averaged across sections from n = 3-5 mice/group.

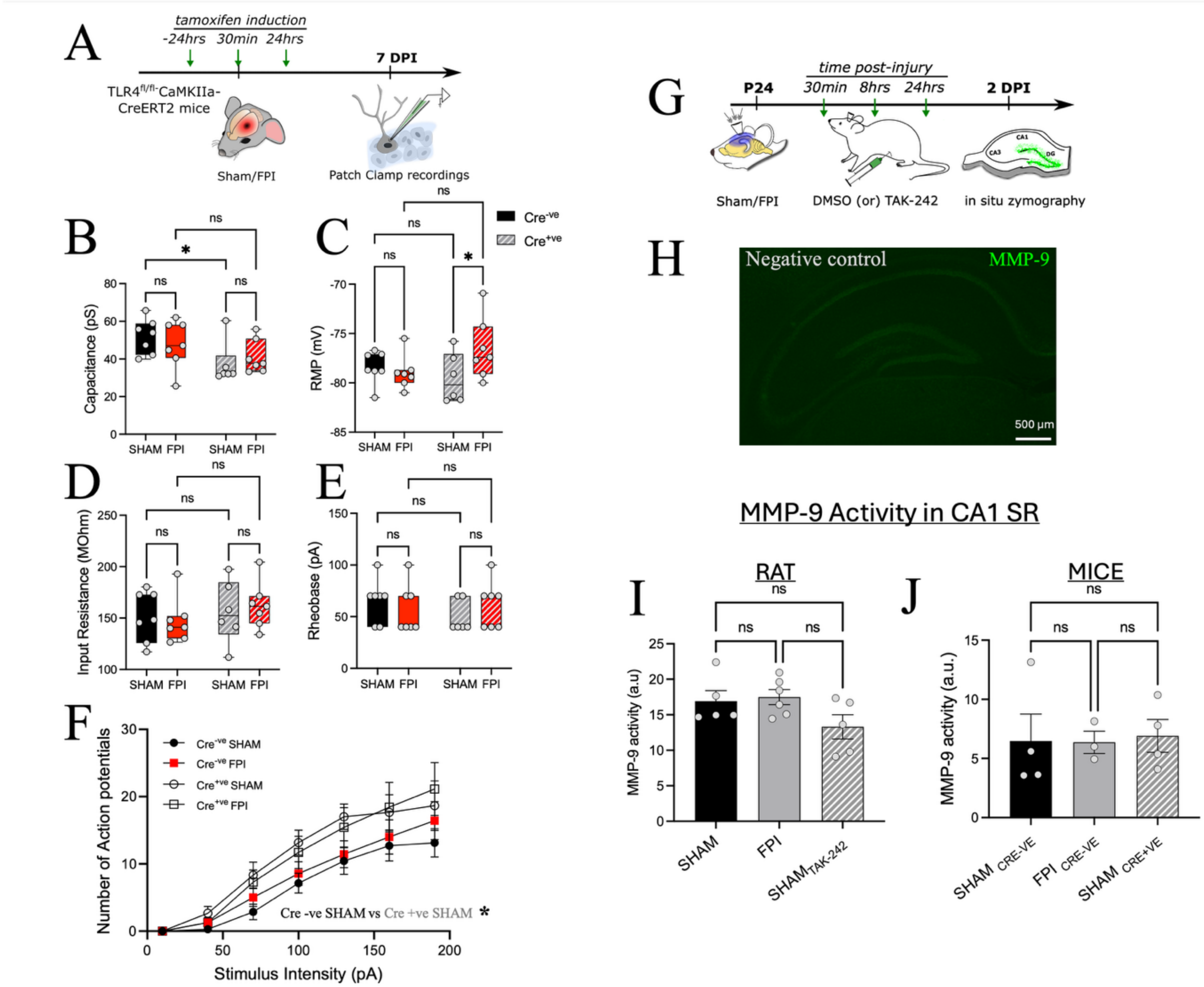

Supplementary Figure 4: *Effects of selective TLR4 deletion in glutamatergic neurons on DGC properties.* A) Experimental timeline for examining active and passive properties of DGCs from TLR4$^{fl/fl}$: CaMKIIa-CreERT2 mice. B-F) Summary plots of active and passive properties including capacitance (B), resting membrane potential (C), input resistance (D), Rheobase (E) and spike frequency (F) in Cre^{-ve} and Cre^{+ve} sham and FPI mice. Note the effect of genotype on cell capacitance and firing frequency in sham injured mice. Also note that the resting membrane potential is significantly depolarized Cre^{+ve} mice after injury, but no differences are seen in Cre^{-ve} mice. Data points in panels B-E represent individual cells (n = 7 cells from 3 mice/group). G)

Experimental timeline for *in situ* zymography experiments. H) Representative images from slices incubated in MMP-9 blocker SB-3CT that served as negative controls. I-J) Quantification of DQ-fluorescence showing CA1 Str. Radiatum intensity is unaltered by injury, treatments or genotype. * indicates $P<0.05$ by TW-ANOVA with Šídáks multiple comparisons post hoc tests (B-F) and OW-ANOVA (I-J). Data points in panel I and J represent individual animals (n = 5-6 rats/group, and 3-4 mice / group, respectively).

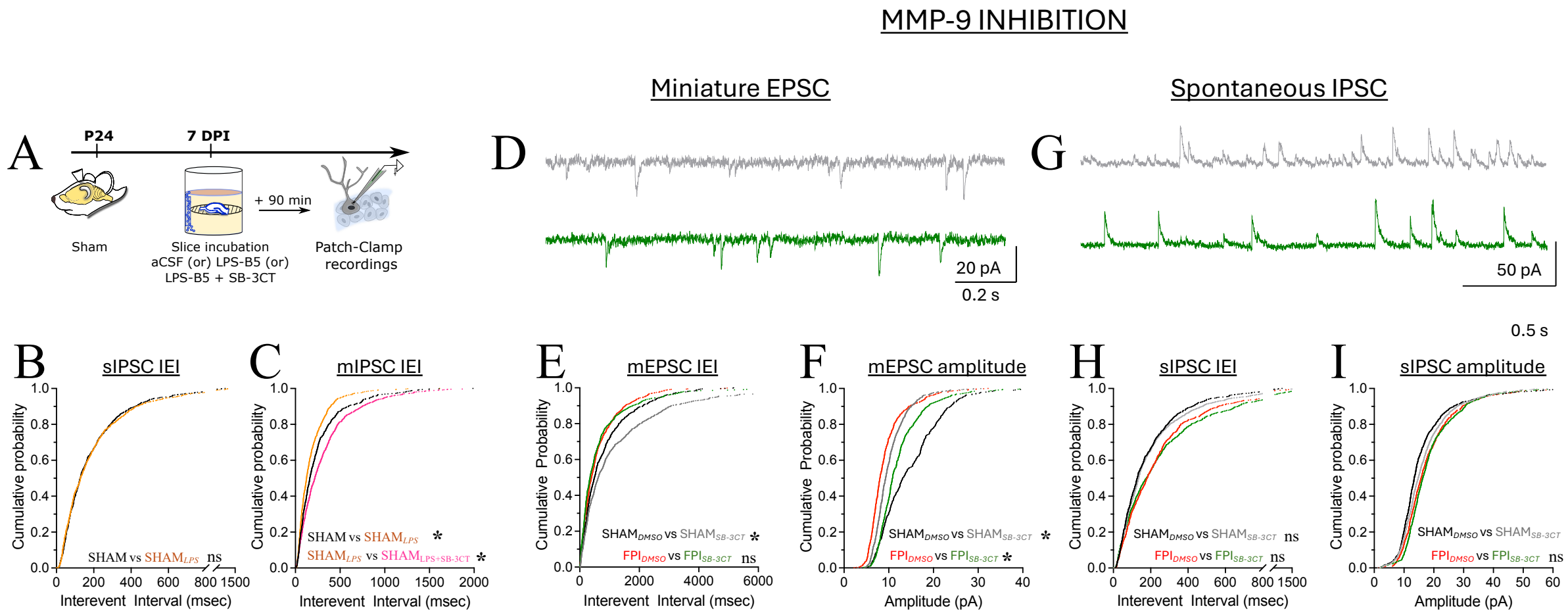

Supplementary Figure 5: ***MMP-9 inhibition modulates mEPSC amplitude without rescuing changes in sIPSC frequency or amplitude.*** A) Experimental timeline for examining changes in synaptic inputs to DGCs in acute rat slices after LPS incubation. B-C) Cumulative probability plots of granule cell sIPSC (B) and mIPSC (C) interevent intervals in sham, LPS-B5 incubated (orange) and LPS-B5+SB-3CT (magenta) co-incubated slices. Note that while LPS-B5 incubation did not alter sIPSC, mIPSC interevent intervals were significantly reduced. Co-incubation of slices in MMP-9 blocker SB-3CT prevented this change. D) Representative granule cell mEPSC traces from SB-3CT treated sham (grey) and injured rats (green). E-F) Cumulative probability plots of mEPSC interevent intervals (E) and amplitude (F) in SB-3CT treated sham/injured rats. G) Representative granule cell sIPSC recordings from SB-3CT treated sham (grey) and injured rats (green). H-I) Cumulative probability plots of mEPSC interevent intervals (H) and amplitude (I) between SB-3CT treated sham/injured rats. n = 8 cells from 4 rats/group (A-C), 7-9 cells from 3 rats/group (D-F) and 8-13 cells from 3 rats/group (G-I) *indicates P<0.05 by Holm-Bonferroni corrected K.S. test.

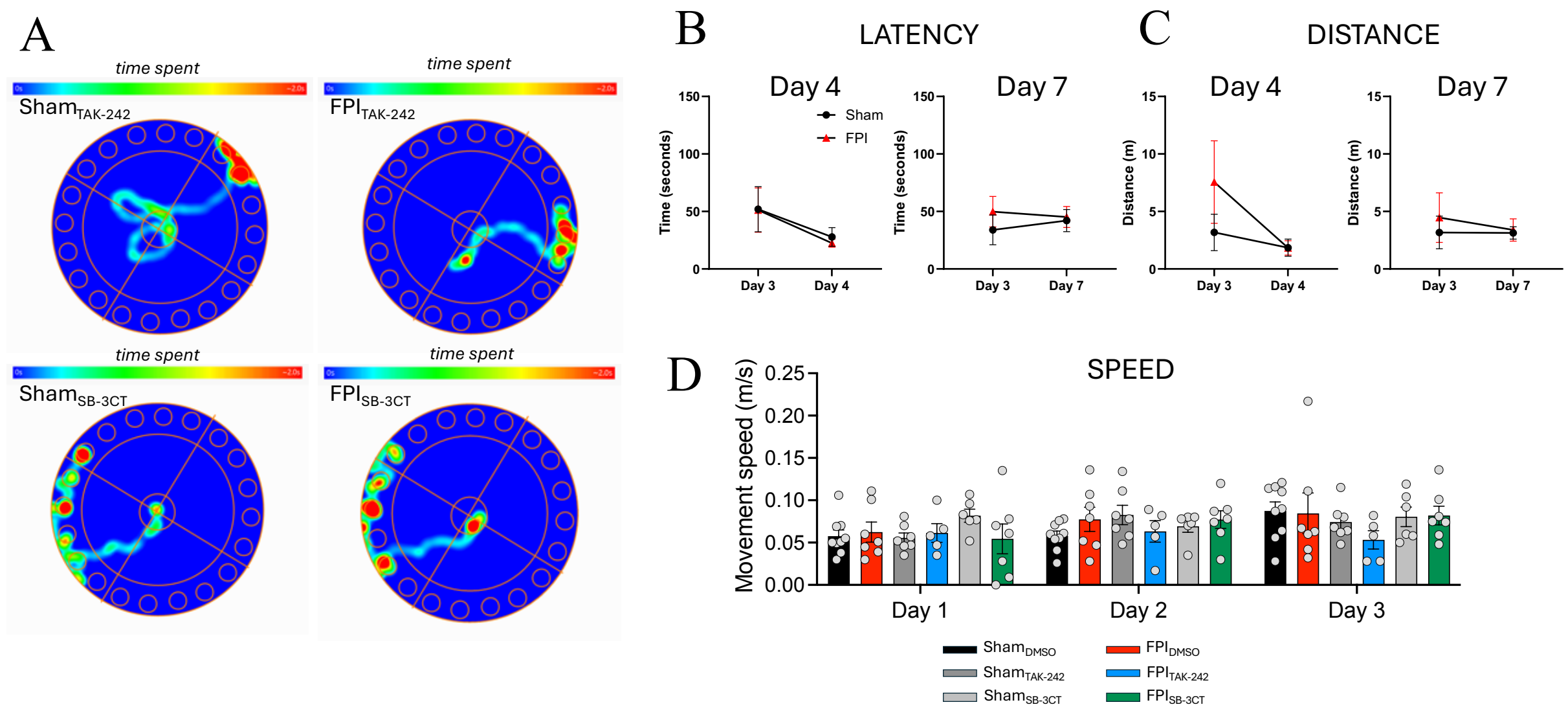

Supplementary Figure 6: ***Treatment with Tak-242 or SB-3CT mitigates Spatial learning deficits in brain injured rats.*** A) Representative heat plots showing trajectory of drug-treated rats in the Barnes maze during task on day 2. B-C) Summary plot of latency to escape (B) and distance travelled (C) on Barnes maze spatial navigation task on day 4 or day 7 in untreated sham and injured rats. D) Summary plot of speed of movement across the training days between the vehicle and drug-treated sham and injured animals showed no injury or training day differences. n = 6-10 rats/group. Data points in panel D represent individual animals.

TABLE 1:

Spontaneous EPSC							
	**Interevent intervals**			**Amplitude**			
	**Mean (ms)**	**Median**	**Interquartile range**	**Mean (pA)**	**Median**	**Interquartile range**	***n* (cells)**
$SHAM_{DMSO}$	336.9	216.4	86.05 - 429.9	13.16	12.20	9.99-15.36	8
FPI_{DMSO}	225.3	144.8	64.60 - 307.4	11.23	10.31	8.06-13.42	8
$SHAM_{TAK-242}$	221.8	143.6	65.74 - 285.6	13.20	12.23	9.64-15.77	9
$FPI_{TAK-242}$	321.9	204.2	95.08 - 420.2	13.82	12.49	9.80-16.20	8
$SHAM_{LPS-B5}$	265.3	157.3	69.18 - 330.4	17.86	17.29	14.06-21.09	8
$SHAM_{LPS-B5+SB-3CT}$	339.7	230.9	94.53 - 434.6	15.90	14.66	11.53-18.96	9
$SHAM_{SB-3CT}$	311.3	203.5	81.18 - 396.8	12.45	11.38	9.22-14.57	10
FPI_{SB-3CT}	495.6	303.8	119.8 - 632.3	12.60	11.64	9.29-14.88	9
Spontaneous IPSC							
$SHAM_{DMSO}$	190.3	128.1	65.35 - 242.4	16.66	13.78	10.81-19.21	8
FPI_{DMSO}	256.6	181.1	83.78 - 320.4	18.24	15.74	11.94-21.70	8
$SHAM_{TAK-242}$	174.2	102.4	50.75 - 199.2	14.79	12.98	10.27-16.85	8
$FPI_{TAK-242}$	200.9	119.7	61.08 - 252	19.95	14.18	10.58-23.23	7
$SHAM_{LPS-B5}$	197.8	128.6	63.33 - 247.5	15.56	13.52	10.99-17.58	8
$SHAM_{LPS-B5+SB-3CT}$	294.3	206.4	92.83 - 395.6	18.01	16.17	12.30-21.37	10
$SHAM_{SB-3CT}$	208.3	135.9	69.60 - 255.9	16.96	14.77	11.16-20.12	13
FPI_{SB-3CT}	288.3	177.2	73.75 - 361.9	18.71	16.28	12.78-21.90	8
**TLR4fl/fl-CaMKIIa-CreERT2 mice – Spontaneous EPSC**							
	**Mean (sec)**	**Median**	**Interquartile range**	**Mean (pA)**	**Median**	**Interquartile range**	***n* (cells)**
Cre^{-ve} SHAM	2.154	1.291	0.45-2.74	13.42	11.65	10.03-14	7
Cre^{-ve} FPI	1.234	0.721	0.31-1.79	11.29	10.36	8.62-12.73	7
Cre^{-ve} FPI_{SB-3CT}	1.734	0.997	0.36-2.24	10.05	9.222	7.57-11.43	7
Cre^{+ve} SHAM	2.223	1.271	0.49-3.10	12.01	11.19	8.90-13.62	6
Cre^{+ve} FPI	1.904	1.203	0.41-2.46	11.27	10.42	8.46-12.65	7

Table 1. Descriptive statistics dentate gyrus cells (DGCs) physiology following traumatic brain injury. Summary of descriptive statistics of interevent intervals and amplitude of spontaneous excitatory and inhibitory postsynaptic currents recorded from DGCs. n represents cells recorded from 3-4 animals/group.

TABLE 2:

Miniature EPSC							
	**Interevent intervals**			**Amplitude**			
	**Mean (ms)**	**Median**	**Interquartile range**	**Mean (pA)**	**Median**	**Interquartile range**	***n* (cells)**
$SHAM_{DMSO}$	824.8	496.1	208.1 - 1096	14.16	12.32	8.90-17.91	7
FPI_{DMSO}	642.4	396.4	158.2 - 858.6	9.28	8.02	6.55-10.55	7
$SHAM_{TAK\text{-}242}$	615.2	325.4	130 - 749.2	12.24	10.54	8.15-15.13	8
$FPI_{TAK\text{-}242}$	1648	621.2	234.5 - 1959	13.82	12.49	9.80-16.20	8
$SHAM_{LPS\text{-}B5}$	726.5	467.7	189.6 - 982.4	10.04	9.219	7.37-11.74	6
$SHAM_{LPS\text{-}B5+SB\text{-}3CT}$	1014	551.1	197.5 - 1272	12.75	11.23	9.53-14.44	10
$SHAM_{SB\text{-}3CT}$	1313	624.1	243.6 - 1660	10.22	9.30	7.63-11.82	9
$FPI_{SB\text{-}3CT}$	710.5	346.9	146.9 - 784.7	12.36	10.85	8.74-14.04	7
Miniature IPSC							
	**Interevent intervals**			**Amplitude**			
	**Mean (ms)**	**Median**	**Interquartile range**	**Mean (pA)**	**Median**	**Interquartile range**	***n* (cells)**
$SHAM_{DMSO}$	254.7	161.2	77.05 - 318.9	15.72	14.15	10.99-18.58	7
FPI_{DMSO}	251	171	86.30 - 336.7	17.68	15.20	11.58-20.72	7
$SHAM_{TAK\text{-}242}$	217.6	150.1	74.63 - 286.1	15.14	13.72	10.54-18.27	7
$FPI_{TAK\text{-}242}$	251	143	64.70 - 300.6	13.75	12.23	9.26-16.35	6
$SHAM_{LPS\text{-}B5}$	183.8	124.9	60.13 - 244.1	14.06	12.56	10.04-16.53	6
$SHAM_{LPS\text{-}B5+SB\text{-}3CT}$	321.6	216.9	90.60 - 420	18.05	16.33	13.19-21.49	10
$SHAM_{SB\text{-}3CT}$	218.1	140	63.63 - 287.2	15.95	14.31	11.41-18.37	11
$FPI_{SB\text{-}3CT}$	366.4	208.1	93.60 - 462.2	16.67	14.90	11.47-19.80	7

Table 2. Descriptive statistics dentate gyrus cells (DGCs) physiology following traumatic brain injury. Summary of descriptive statistics of interevent intervals and amplitude of miniature excitatory and inhibitory postsynaptic currents recorded from DGCs. n represents cells recorded from 3-4 animals/group.

TABLE 3: *Holm-Šídák Adjusted P-values*

	Excitatory currents			
	**Spontaneous**		**Miniature**	
	IEI	amplitude	IEI	amplitude
SHAM$_{DMSO}$ (vs) FPI$_{DMSO}$	*P*<0.0001; d = 0.344	*P*<0.0001; d = 0.410	*P*=0.0027; d =0.217	*P*<0.0001; d = 0.853
FPI$_{DMSO}$ (vs) FPI$_{TAK-242}$	*P*<0.0001; d = 0.615	*P*<0.0001; d =0.502	*P*<0.0001; d =0.543	*P*<0.0001; d = 0.889
SHAM$_{DMSO}$ (vs) SHAM$_{TAK-242}$	*P*<0.0001; d = 0.354	*P=0.5297*; d = 0.008	*P*<0.0001; d =0.239	*P*<0.0001; d = 0.301
SHAM$_{DMSO}$ (vs) Sham$_{LPS}$	*P*<0.0001; d = 0.202	*P*<0.0001; d = 0.766	*P*=0.2463*;* d = 0.113	*P*<0.0001; d = 0.788
Sham$_{LPS}$ (vs) Sham$_{LPS + SB-3CT}$	*P*<0.0001; d = 0.216	*P*<0.0001; d = 0.325	*P*=0.0012*;* d = 0.237	*P*<0.0001; d = 0.538
SHAM$_{DMSO}$ (vs) Sham$_{SB-3CT}$	*P* =0.6476*;* d = 0.069	*P* =0.0002*;* d = 0.147	*P* =0.0001*;* d = 0.311	*P*<0.0001; d = 0.738
FPI$_{DMSO}$ (vs) FPI$_{SB-3CT}$	*P*<0.0001; d = 0.601	*P*<0.0001; d = 0.301	*P* =0.1108*;* d = 0.073	*P*<0.0001; d = 0.620
	**Inhibitory currents**			
SHAM$_{DMSO}$ (vs) FPI$_{DMSO}$	*P*<0.0001; d = 0.290	*P*<0.0001; d = 0.157	*P*=0.5139; d = 0.013	*P*=0.0044; d = 0.220
FPI$_{DMSO}$ (vs) FPI$_{TAK-242}$	*P*<0.0001; d = 0.221	*P*<0.0001; d = 0.112	*P*=0.0748; d =0.000	*P*<0.0001; d = 0.473
SHAM$_{DMSO}$ (vs) SHAM$_{TAK-242}$	*P*=0.0005; d = 0.072	*P*=0.0037; d = 0.206	*P*=0.5139; d = 0.419	*P*=0.3 808; d = 0.077
SHAM$_{DMSO}$ (vs) Sham$_{LPS}$	*P*=0.8642; d = 0.037	*P*=0.0222; d = 0.107	*P*=0.0004; d = 0.293	*P*<0.0001; d =0.226
Sham$_{LPS}$ (vs) Sham$_{LPS + SB-3CT}$	*P*<0.0001; d =0.378	*P*<0.0001; d = 0.246	*P*<0.0001; d = 0.468	*P*<0.0001; d = 0.549
SHAM$_{DMSO}$ (vs) Sham$_{SB-3CT}$	*P*=0.4758; d = 0.085	*P*<0.0001; d =0.138	*P*=0.0542; d = 0.146	*P*=0.2826; d =0.030
FPI$_{DMSO}$ (vs) FPI$_{SB-3CT}$	*P*=0.4287; d = 0.098	*P*=0.0597; d = 0.048	*P*=0.0012; d = 0.299	*P*=0.5412; d = 0.113
**TLR4fl/fl-CaMKIIa-CreERT2 mice – Spontaneous Excitatory currents**				
Cre^{-ve} SHAM (vs) Cre^{-ve} FPI	*P*<0.0001; d = 0.420	*P*<0.0001; d = 0.311		
Cre^{+ve} SHAM (vs) Cre^{+ve} FPI	*P*=0.1109; d = 0.131	*P*=0.0008; d = 0.157		
Cre^{-ve} FPI vs Cre^{-ve} FPI$_{SB-3CT}$	*P*<0.0001; d = 0.283	*P*<0.0001; d = 0.303		

Table 3. Statistical analysis of synaptic inputs to dentate gyrus cells (DGCs) following traumatic brain injury. Summary of Holm-Bonferroni corrected *P*-values (Kolmogorov-Smirnov tests) and Cohen's *d* effect sizes for comparisons of synaptic inputs to DGCs between sham and FPI groups. Animals received either vehicle or the TLR4 inhibitor TAK-242. Parameters include interevent intervals and amplitudes for both spontaneous and miniature excitatory postsynaptic currents (sEPSC/mEPSC) and inhibitory postsynaptic currents (sIPSC/mIPSC).

TABLE4:

	**10min**	**60min**	**120min**	**180min**
$SHAM_{DMSO}$	157.71±6.58	144.00±4.30	146.78±4.82	145.15±7.03
FPI_{DMSO}	130.23±6.92	116.69±3.84	112.84±4.20	110.36±5.31
$SHAM_{TAK-242}$	150.12±7.95	133.00±5.58	124.33±8.60	116.11±6.98
$FPI_{TAK-242}$	149.14±8.95	143.91±4.66	140.32±4.02	141.21±5.00
$SHAM_{SB-3CT}$	164.56±5.67	159.66±2.40	156.98±8.57	154.61±7.70
FPI_{SB-3CT}	166.98±10.3	157.60±10	146.85±6.61	134.76±8.62

Table 4. Maintenance of long-term potentiation (LTP) in the hippocampal dentate gyrus post-injury. Normalized slope values (mean ± SEM) measured at 10, 60, 120, and 180 min following theta burst stimulation (TBS). Measurements were recorded one-week post-injury in sham and FPI rats treated with vehicle (DMSO), a TLR4 inhibitor (TAK-242), or an MMP-9 inhibitor (SB-3CT).

TABLE 5:

		Day1		Day2		Day3	
**Group**	*n* (rats)	Latency (seconds)	Distance (meters)	Latency (seconds)	Distance (meters)	Latency (seconds)	Distance (meters)
$SHAM_{DMSO}$	10	61.36±11.11	3.32±0.87	49.19±9.30	2.83±0.36	41.18±10.78	3.17±1.00
FPI_{DMSO}	10	113.1±16.68	7.20±1.67	87.06±10.64	9.07±1.72	50.21±10.34	5.39±1.80
$SHAM_{TAK-242}$	7	69.30±19.92	3.14±0.58	82.90±25.52	6.06±1.41	67.74±21.15	4.84±1.01
$FPI_{TAK-242}$	7	102.2±24.2	5.25±1.10	40.26±11.01	2.76±0.46	58.04±16.63	3.53±1.18
$SHAM_{SB-3CT}$	6	82.58±24.05	10.4±2.30	42.12±10.34	3.00±0.84	62.50±6.84	4.68±0.89
FPI_{SB-3CT}	7	67.86±18.33	5.43±2.67	46.66±4.97	3.46±0.40	51.73±22.18	4.51±2.29

Table 5. Spatial memory performance in the Barnes maze task one month after brain injury. Mean latency (seconds) to reach the escape hole and total distance traveled (meters) by sham and FPI rats on training days 1–3. Testing was conducted one-month post-injury and values are given as mean ± SEM. Treatment groups received vehicle (DMSO), TLR4 inhibitor (TAK-242), or MMP-9 inhibitor (SB-3CT) immediately following injury.
